# Indoor Content Delivery Solution for a Museum Based on BLE Beacons

**DOI:** 10.3390/s23177403

**Published:** 2023-08-25

**Authors:** David Verde, Luís Romero, Pedro Miguel Faria, Sara Paiva

**Affiliations:** ADiT-Lab from Instituto Politécnico de Viana do Castelo, Rua Escola Industrial e Comercial de Nun’Álvares, n.° 34, 4900-367 Viana do Castelo, Portugal; romero@estg.ipvc.pt (L.R.); pfaria@estg.ipvc.pt (P.M.F.); sara.paiva@estg.ipvc.pt (S.P.)

**Keywords:** indoor-location, BLE beacons, path loss, RSSI signal, museums

## Abstract

The digital transformation advancement enables multiple areas to provide modern services to their users. Culture is one of the areas that can benefit from these advances, more specifically museums, by presenting many benefits and the most emergent technologies to the visitors. This paper presents an indoor location system and content delivery solution, based on Bluetooth Low Energy Beacons, that enable visitors to walk freely inside the museum and receive augmented reality content based on the acquired position, which is done using the Received Signal Strength Indicator (RSSI). The solution presented in this paper was created for the Foz Côa Museum in Portugal and was tested in the real environment. A detailed study was carried out to analyze the RSSI under four different scenarios, and detection tests were carried out that allowed us to measure the accuracy of the room identification, which is needed for proper content delivery. Of the 89 positions tested in the four scenarios, 70% of the received signals were correctly received throughout the entire duration of the tests, 20% were received in an intermittent way, 4% were never detected and 6% of unwanted beacons were detected. The signal detection is fundamental for the correct room identification, which was performed with 96% accuracy. Thus, we verified that this technology is suitable for the proposed solution.

## 1. Introduction

Museums play an essential role in preserving and transmitting local culture [1,2]. With the careful preservation of documentation and artifacts, culture can be recorded and remembered regardless of its future. The past can be learned by everyone in order to prepare the future, and cultural backgrounds can be shared across generations. It is important to promote and develop museum environments where visitors are able to access space information and therefore have a more enriching visiting experience [3].

Multiple challenges arise when referring to the indoor environment of a museum. One of them is the unavailability of a guide when required, which might lead to the visitor obtaining less information about certain artifacts and/or the eventual need for extra payment to have access to a more individualized experience [4]. Also, it is well-known by museum staff that visitors often become bored during the visit, as a consequence of the difficulty of gaining the visitor’s attention throughout the entire route, which represents a challenge in these environments, especially if visitors are children [5]. Understanding the users’ preferences and position is therefore essential to overcome the difficulty of engaging the visitor throughout the entire duration of the visit. To address this, several applications exist that assist museums in obtaining the position of the visitors to provide them with a guided experience. However, most solutions act explicitly, requiring the user to go to a checkpoint or scan a QR code, which makes them have to follow a predetermined route and lose the sense of freedom to conduct the visit as they wish. Allowing the visitor to walk freely through the museum and explore it at their own pace is a way to increase their interest in the visit. Another factor that contributes to this increase is the greater interaction of the environment with the visitor, which is possible from the moment the visitor is able to explore the surroundings with contextual content that is automatically presented to him depending on the context in which he is placed.

Therefore, the importance of an indoor solution that automatically perceives the user’s position is fundamental in this type of scenario. This paper presents an indoor-location content-delivery system solution for the Foz-Côa Museum in Portugal, using Bluetooth Low Energy (BLE) beacons. The Houdini project emerged from the museum’s intention to improve the visitor experience, and transform it into something interactive and dynamic, presenting additional information about the artifacts and exposition rooms (hereafter simply referred to as rooms), without the need for human intervention.

This paper presents a case study conducted at a museum, where specific localization functionalities were analyzed and designed to cater to the museum’s unique requirements. The insights gained from this study can be readily applied and extended to enhance localization capabilities in various other indoor environments, even those with distinct purposes and settings. The main contributions of this paper include the real implementation of an application that combines augmented reality technology with an indoor-location system based on BLE beacons. A technological architecture proposal for an indoor-location-based solution using Bluetooth Low Energy Beacons. Test scenarios in a real environment regarding the accuracy of the detection of the beacon using Received Signal Strength Indicator (RSSI) -based technology. An analysis of the accuracy of the room identification inside the museum and a proposal of the beacons distribution in a real case scenario considering the lowest costs.

The rest of this paper is organized as follows. Section 2 presents the review of related work. Section 3 introduces the main features of beacons as a way to support indoor locations as well as the ranging technique used for the proximity estimation. Section 4 introduces the proposed solution architecture specifying all the components and its characteristics. Section 5 presents the mobile application architecture that makes part of the global proposed solution. Section 6 explains the algorithm functionality in relation to beacon localization. Section 7 describes the integration of augmented reality technology with beacon-based positioning and explains how these components synergistically contribute to the proposed solution. Section 8 presents the tests carried out as well as the experimental results obtained. Before the conclusions are presented, Section 9 discusses the best beacon positioning techniques to consider in similar situations.

## 2. Related Work

With the evolution of technology and the constant growth of smartphone and tablet usage, the creation of sophisticated and more accurate real-time indoor-location systems became possible [6,7]. One of the most used technology for this purpose is BLE beacons. These BLE indoor-location systems are RSS-based and popular due to their accuracy, low cost, and low complexity [8,9].

There are several domain areas that can benefit from these technological advances, namely, tourism, healthcare, and industry. An example of a healthcare application is presented in [10], where authors focus on an indoor solution, beacon-based system for a hospital environment. Their main goal is to provide hospital/nursing home staff the ability to track staff, patients, visitors, and equipment and quickly locate their position, in order to prevent unauthorized departure, quickly locate needed equipment and machinery, and increase security in the hospital.

Another suitable application domain is the industrial area. The authors in [11] propose to apply a WiFi and Bluetooth (BLE 4.0) tracking system, specifically for industrial applications, using portable mobile devices. The study revealed that Bluetooth tracking, with a slight cost trade-off, provides improved precision and allows the user to follow the position at a typical speed. Larger changes in the environment, such as the installation or removal of machine parts, will, nevertheless, have an impact on the system’s confidence. As a result, a monthly update in fingerprints to maintain confidence levels is unavoidable.

Several museums are also adopting BLE technology [12]. In [13], authors describe an indoor localization system that improves the museum visitor experience. The suggested system is built on the proximity and localization capabilities of BLE beacons, as well as an RSS-based approach that automatically presents users with cultural information connected to nearby artworks. This location system was developed on an Android application that not only offers content information to the user, but also gathers relevant analytic data about each visit and recommends material to the user. Because of their success, the authors concluded that BLE beacons are a potential option for an interactive smart museum and, as such, should be employed often in these circumstances.

The scientific community is also focusing on specific studies to discover patterns of human behavior utilizing real-time location technologies [14]. In [15], the authors study behaviors using indoor-location data. They emphasize four areas where these technologies are being used effectively: health status monitoring, consumer habits, developmental behavior, and workplace safety/efficiency. The collected data were divided into four categories: dwell duration, activity level, trajectory, and proximity.

In order to increase the accuracy of RSSI-based indoor-location systems, several articles research the use of machine learning and neural networks for this purpose [16,17]. In [18], the authors conducted a study on using BLE beacons and Feed Forward Neural Networks (FFNN) for indoor localization in IoT applications. They trained a FFNN using signal strength values from thirteen BLE iBeacon nodes in an indoor environment. The FFNN achieved an 86% accuracy in classifying the correct zone, suggesting that FFNNs could be used for implementing location-based IoT applications.

In addition to the mentioned application domains, indoor location systems founded upon BLE beacons present significant implications for critical areas such as emergency management, intelligent energy management, advanced HVAC controls, and accurate occupancy detection. These technologies have the potential to transform spatial tracking, resource allocation, and operational efficiency in various indoor environments, thereby fostering advancements in safety, sustainability, and overall system optimization.

The significance of reliable occupancy estimation in emergency management is explored in [19]. BLE technology is evaluated as a viable solution for indoor occupancy detection, leveraging beacons within buildings to track user locations. The study employs three machine learning techniques: k-nearest neighbors, logistic regression, and support vector machines; to detect occupants in specific areas of an office space. The experimental results demonstrate the potential of combining BLE with machine learning for accurate occupancy estimation, providing a promising basis for further research in this field.

Regarding intelligent energy management, an IoT-based plug load management system designed to reduce energy consumption in commercial buildings named Plug-Mate is presented in [20]. It considers occupancy-driven automation, advanced plug load identification, and personalized user preferences. A 5-month field study in an office space showed average energy savings of 51.7% across different plug load types, with a 7.5% reduction in overall building energy use.

Authors in [21] introduced a cost-effective solution for reducing HVAC energy consumption in commercial buildings. By leveraging existing WiFi infrastructure and visitors’ smartphones with WiFi connectivity, it enables fine-grained occupancy-based HVAC actuation. The system achieves accurate occupancy detection in office spaces 86% of the time, with only 6.2% false negative errors. Despite some inaccuracies due to smartphone power management, the solution successfully actuates 23% of HVAC zones within a commercial building, resulting in significant HVAC electrical energy savings of 17.8%.

A minimal sensing strategy with comprehensive sensor data is used in [22] to predict occupancy in various building spaces. The proposed feature selection algorithm outperforms conventional methods, achieving higher model performance with fewer sensing requirements. Crucial features include indoor CO_2_ levels and Wi-Fi connected devices, and the best model performances are attained using Bi-GRU for offices and lecture rooms, and GRU for libraries.

When it comes to deciding which indoor location system should be used, the target environment and the solution specificity must be considered. Several research exists in that direction [23,24,25]. Multiple technologies have been proposed in the past to perform indoor localization, many of which have demonstrated good localization performance for different use cases. Regarding this study, multiple articles [26,27,28,29], aim to compare technologies such as RFID, Ultrasonic Sensors, Bluetooth Low Energy, Ultra-Wide-band, and Wi-Fi in order to identify occupancy patterns and profiles in indoor spaces. The basic specifications of some indoor positioning technologies are presented in Table 1.

RFID offers reliable identification but requires occupants to carry RFID tags, which might be inconvenient and impact user acceptance. Ultrasonic sensors can detect occupancy accurately, but their installation and maintenance can be burdensome and costly. UWB [30] offers high data transfer rates, enabling fast and efficient wireless communication, and provides precise indoor positioning and tracking capabilities; however, UWB devices may have higher power consumption and implementation complexity compared to other solutions and there is a possibility of interference with other wireless technologies. In contrast, BLE proves promising due to its ability to provide location data without the need for additional tags or devices. BLE-enabled smartphones or devices carried by occupants can serve as beacons, making it a cost-effective and non-intrusive solution. Additionally, BLE benefits from wide adoption, making it more accessible for implementation. Wi-Fi, while widely available, may not provide the same level of location accuracy as BLE due to its design as a network connectivity technology. However, its potential for occupancy detection can still be leveraged to some extent.

Overall, these studies conclude that a scalable BLE approach is a favorable choice for identifying occupancy patterns and profiles in indoor spaces. Due to this favoritism and our scenario environment constitution, the use BLE technology was considered because its non-intrusive nature, cost-effectiveness, and utilization of existing devices make it a viable and efficient solution for modern buildings and applications.

This paper describes the use of BLE beacons as part of a cyber-physical component that communicates with a mobile app that makes use of augmented reality. Beacons are the only source of data to determine the location of the user inside the museum, which is necessary to deliver the right content. Hence, the importance of studying the accuracy of the beacon’s detection.

## 3. Beacons to Support Indoor Localization

In this section, the main features of beacons as a way to support indoor localization are described as well as the ranging technique used for the proximity estimation.

### 3.1. BLE Beacons

Beacon devices are small-size, wireless transmitters that use BLE technology to send radio signals to all nearby devices that support Bluetooth signals. They are currently one of the most used location technologies for both indoor and outdoor environments. Basically, they connect and transmit information to nearby devices making location-based searches easier and more accurate.

Allowing us to locate devices in a specific environment is the beacon’s main objective. These devices might represent users, equipment, or other objects, and according to the detected beacon, which corresponds to a given place, information, alerts, and promotions can be presented to the user.

**Figure 1:**
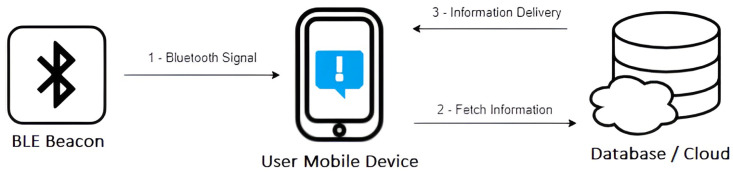
Beacon technology architecture.

A beacon device is quite simple, being composed of a Central Process Unit (CPU), a Bluetooth-based radio signal emitter, and batteries. It works by periodically broadcasting its unique identifier and some more data packets to nearby Bluetooth-compatible devices, then these devices fetch specific data according to the identifier received on a database, as depicted in Figure 1. The identifier is a unique ID number that devices recognize as unique to the corresponding beacon. Each identifier or group of identifiers represents a concrete place inside a specific environment. This identifier is received by the nearest devices, which are usually mobile ones (smartphones and tablets), which allows us to identify the position of the devices.

As with all technologies, beacons have both advantages and disadvantages [31]. In each scenario, it is necessary to decide if this is a suitable technology. Some of the advantages include:

- Beacon devices are affordable and easy to install and setup, which makes them low risk and a high potential return on investment.
- Beacon technology has great location precision and can be used in almost all environments.
- Beacon usage increases the user’s engagement in the environment and enables a boost in the application area.

In terms of disadvantages, the following can be highlighted:

- Most digital users are not comfortable with companies having access to their location and path data, which can lead to not using location-based applications.
- Beacon technology is limited to BLE signals, therefore, if a customer does not have Bluetooth enabled or if the device being used is not compatible with Bluetooth, beacon technology will not be able to detect them.
- Beacon’s location functionality is only possible if a certain application is previously installed in order for the beacon technology to communicate with the user’s device. Many users may not install the app.

### 3.2. RSSI

RSSI stands for **Received Signal Strength Indicator** and it is one of the most used location techniques using radio waves. In beacon’s context, RSSI represents the strength of beacon’s signal in the receiving device. The signal strength depends on the distance between the beacon and the receiver device and from the broadcasting/transmission power (TX) value. For Kontakt beacons, the ones used in the test scenarios presented in this paper, considering a minimum transmission power at 1 m distance, the approximate RSSI is −84 dBm. On the other hand, considering a maximum transmission power at 1 m, the RSSI is approximately −59 dBm. Periodically, beacons send their identifier, transmission power and some more data packets to nearby devices that support BLE signals. The RSSI value can be calculated through the following formula [32], where *n* indicates the path loss index in a specific environment, which is bigger along with the increasing distance, *d* is the distance between the beacon and the receiver device, and $RSS_{1}$ represents the approximate RSSI value at a 1 m distance:

$$RSSI=-10n\log_{10}d+RSS_{1}$$

RSSI is most of the time used to calculate an approximate distance between the receiver device and the beacon. To archive that, the following formula can be used:

$$d=10^{(RSS_{1}-RSSI)/10n}$$

The reduction in power density of an electromagnetic wave, as it propagates through space, due to indoor environmental factors influencing these waves such as absorption, refraction, interference, or diffraction, is called Path Loss [33]. The further away the device is from the beacon, the more unstable the RSSI becomes.

Through the following Path Loss formula, it is possible to calculate the path loss index— *n* value—and then verify if the implementation environment is or is not challenging. The great *n* value obtained the more challenging environment.

$$n=\frac{RSS_{1}-RSSI}{10log(d)}$$

**Figure 2:**
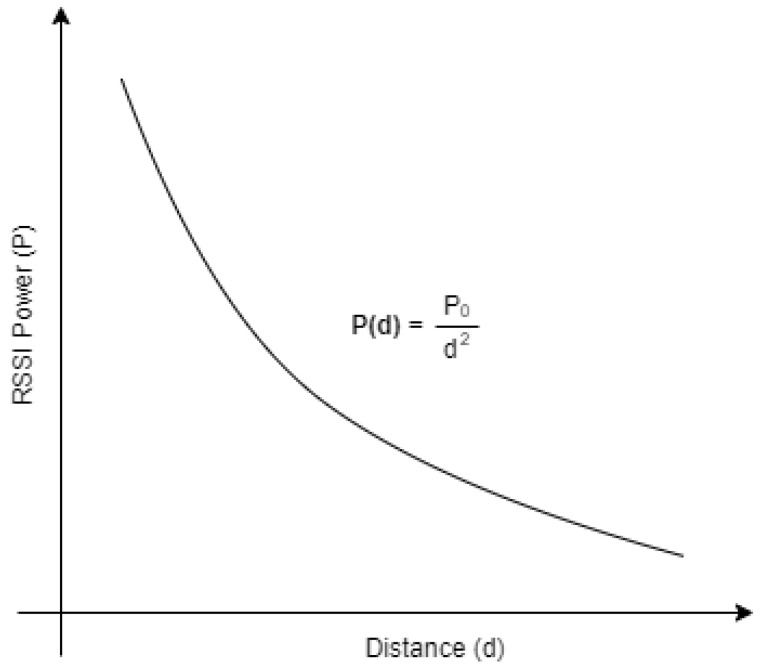
RSSI power curve related to distance.

In a perfect environment, with no interference, the RSSI power intensity should decrease like the square of the distance, as shown in Figure 2. This means that the further the receiver device is from the beacon, the less power it captures.

## 4. Proposed Solution Architecture

### 4.1. General Architecture

**Figure 3:**
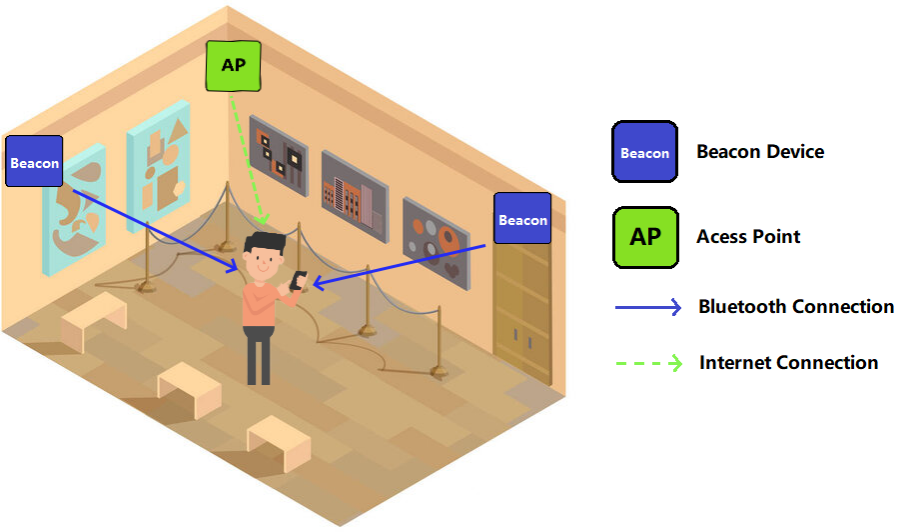
System overview.

Following the requirements defined by the Foz-Côa museum to deliver content-based information to its visitors, the defined system architecture to achieve this purpose is presented in Figure 3. This architecture is composed of three main components:

1. A set of **BLE Beacon** devices that periodically send a signal;
2. The **visitor’s mobile device** that receives information from the beacons and allows us to determine the room where the visitor is located;
3. Multiple **Access Points** distributed by the museum rooms, in order to establish an internet connection with cloud databases and access contents.

In the indoor environment of the museum, beacon devices are used to track the visitor’s location and obtain the maps and contents of a certain room. The load of maps and contents was prioritized in order to optimize the application functionality; the lesser AR maps and AR contents loaded at the same time, the better the application performance will be, especially when the targeted museum is quite extensive. Depending on the environmental characteristics, at least two (or more) beacons per room should be used, one placed at each room’s entrance/exit and the other placed in an inside central room position.

**Figure 4:**
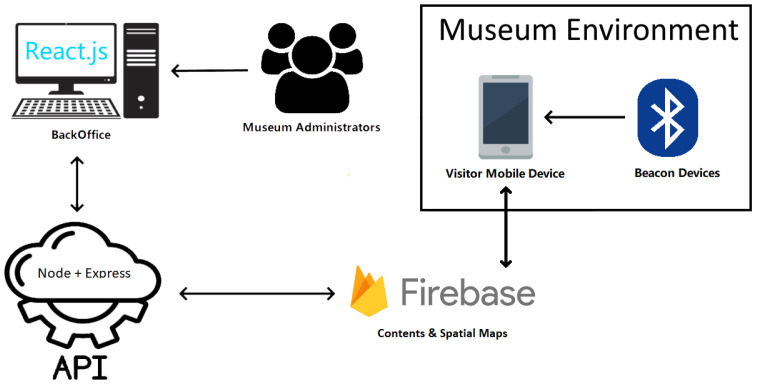
Overall system architecture.

The defined technological architecture is depicted in Figure 4. Inside the museum environment, the visitor’s mobile device is the main component and has the AR application previously installed. The application receives the beacon’s data and calculates the room the user is in, and then AR maps and contents are loaded from a Firebase database. This database is fed by the museum’s administrators through a backoffice developed in *React.js* and an API developed with *Node* and *Express*.

### 4.2. Beacons Specifications

Despite the existence of multiple beacons with different specifications in the market, we choose to test and use the **Anchor Beacon 2**, from Kontakt company, due to their long lifetime, replaceable batteries, and general quality-price relation. These beacons have space for 2 batteries (ER14250—1.2 Ah) that can be replaceable and that last up to 8 years with certain configurations. In terms of connectivity, they are equipped with Bluetooth Low Energy 5.0, with a range of up to 100 m and a transmission power that can be changed from −20 to +4 dBm. Anchor Beacon 2 is small (49 mm × 49 mm × 15 mm) and light (38 g). These specifications are shown in Table 2.

## 5. Mobile Application

**Figure 5:**
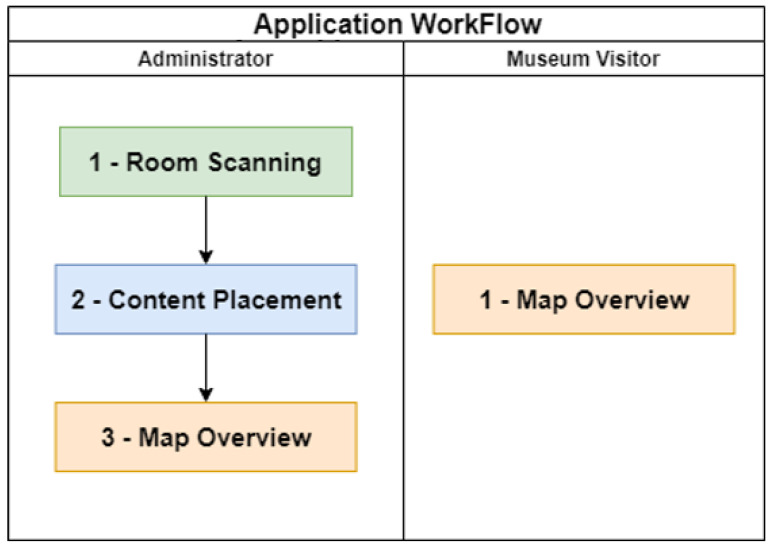
Applications workflow.

Two mobile applications were developed in the ***Unity3D*** game engine using the C-Sharp (C#) language. One of the applications will be used by museum administrators providing the functionality to scan rooms, add digital media content, and preview the augmented reality world. The other application will be used by museum visitors and has the objective of showing them content placed in an augmented reality world. Both applications’ workflow is presented in Figure 5.

The first phase—**Room Scanning**—of the administrator application allows museum administrators to scan indoor spaces, transforming those into virtual maps.

The second phase—**Content Placement**—of the administrator application allows museum administrators to position contents in the virtual maps, creating an augmented reality world.

The third phase—**Map Overview**—is equally destined for museum administrators and visitors. It allows both parts to see the augmented reality world created through the mobile device being used.

**Figure 6:**
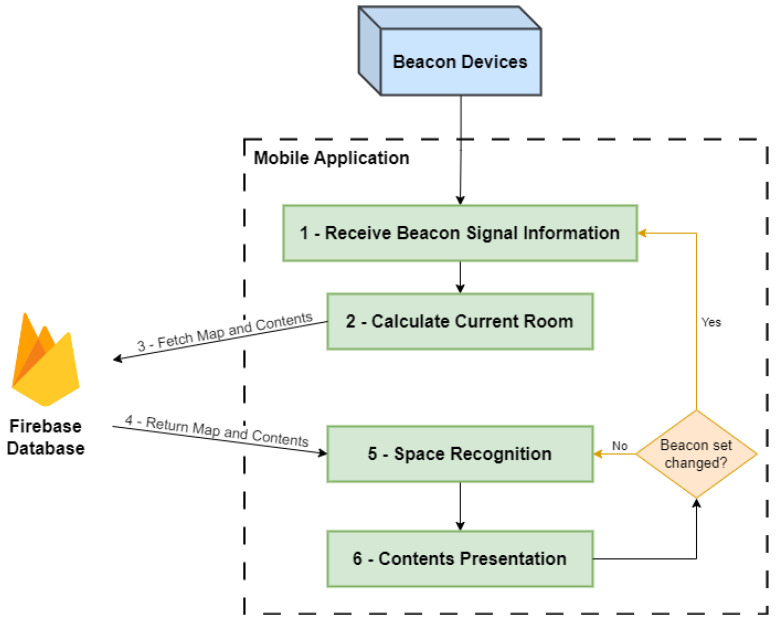
Mobile application architecture.

Figure 6 presents the mobile application workflow. It is composed of the following services and connections:

1. **Collect proximity Beacon Signal**: The visitor’s mobile device is constantly listening for local beacon signals. This is the first step needed to move forward. When a certain beacon’s data are received, the application goes to the next step.
2. **Calculate Current Room**: The previously received beacon’s information is processed to calculate the current room that the visitor is in. One possible scenario is that only one beacon signal is received by the mobile device, which represents the room the visitor is in. If the device, additionally, captures signal information from other rooms, both spaces will be considered in the next step of the process.
3. **Fetch Map and Contents**: After the current room is calculated, a fetch request to a Firebase database is made, in order to obtain that room’s AR map and contents.
4. **Return Map and Contents**: The Firebase database returns the wanted room’s AR map and contents back to the application.
5. **Space Recognition**: Using the AR tool, the space recognition starts, comparing, in real-time, the captured video with the AR map previously fetched.
6. **Contents Presentation**: The application is presented the real-world view overlapped with the AR world. In specific places, the fetched AR contents are shown to the visitor in the mobile application.
7. **Beacon Set Changed**: While presenting the contents the application verifies if the received beacon’s data have changed. If not, the application continues doing the space recognition and showing contents; If it has changed, the new current room is calculated and the loop starts again.

## 6. Beacon Localization Algorithm

With this solution is proposed a simplified Bluetooth Beacon Localization Algorithm for indoor positioning applications, that uses the presence of BLE beacons deployed in an indoor environment, where each beacon represents a distinct room or area. By capturing the nearby Bluetooth packets transmitted by each beacon, we are able to determine the location of a device within the facility. The location is calculated at room level.

### Algorithm Functionality

Considering a previously deployed network of BLE beacons throughout the indoor space of interest and that each one of these devices is configured with a universally unique identifier, allowing it to be associated with a specific room or area. These beacons continuously transmit Bluetooth packets containing their respective identifiers. One room or area can be identified by multiple beacons depending on its size and the beacon’s transmission range.

When a device with our application installed enters the range of these beacons, it listens for and captures the Bluetooth packets being transmitted. The application can identify the beacon’s unique identifier by analyzing the received packets. Based on our predetermined mapping of beacons to rooms, the application can infer the location of the device within the indoor environment.

By considering the beacon as a representative of a specific room, we eliminate the need for complex RSSI-based localization algorithms. Instead, the focus is on detecting the nearby beacons and associating them with the corresponding rooms. This simplification streamlines the system implementation and reduces computational overhead, making it suitable for various indoor positioning scenarios.

The possibility of a user’s device detecting signals from multiple rooms simultaneously has also been considered. To address this situation, our system employs a comprehensive approach, considering all detected rooms as potential locations for the user. As the user’s device is within the range of the beacons in these rooms, it signifies the user’s proximity to those areas, enabling quick access and reducing loading delays. Therefore, to enhance user experience and ensure a seamless navigation process, we adopt a pragmatic solution by loading multiple map rooms detected by the user’s device into the application. By adopting this approach, our system ensures that all relevant map rooms are readily available to the user, allowing them to efficiently access the desired location without experiencing unnecessary waiting times. Moreover, our system emphasizes user-friendliness, prioritizing ease of navigation and minimizing loading delays, thereby enhancing the overall usability of the application.

## 7. Integrating Augmented Reality with Beacon-Based Positioning

Considering the real targeted museum and the need to provide a more immersive, interactive, and contextually relevant experience, we propose integrating augmented reality technology with an indoor localization using beacons. BLE beacon indoor localization and AR technology have a good synergy. A framework that leverages the user’s location information to deliver customized augmented reality content and virtual maps in real time was created. Our application makes use of the previous room location gathered from the beacon’s information as a trigger to download and display AR overlays and digital content specific to the current room.

The proposed application allows the following user experience advancements:

1. **Object-Oriented Information:** As the user moves within the indoor environment, the application uses the device’s localization to precisely determine the room or area in which the user is located. By associating the user’s position with the corresponding room, the application can download and present contextually relevant AR content. This content may include 3D models, images, videos, audio, or multimedia overlays, all tailored to enhance the user’s understanding and engagement within the space.
2. **Smooth Transitions:** The integration of AR technology with BLE beacons’ indoor location enables seamless transitions between physical and augmented realities. As the user moves from one room to another, the system quickly detects the change in location using the beacon technology. This way, the application can automatically fetch the right AR content for the new room, providing a smooth and uninterrupted AR experience without user intervention.
3. **Reduced Processing Power:** Relying on the previously calculated user’s location, our application minimizes the need for computationally intensive algorithms on the user’s device. Instead, the application primarily focuses on downloading and rendering AR content related to the current room. This approach optimizes the app’s performance, reduces processing power consumption, and enhances the overall user experience by maintaining smooth and responsive AR interactions, once again increasing the range of possible users.

## 8. Experimental Results

The performance of the proposed system was tested in several scenarios in a real environment—the Foz Côa Museum, in Portugal. Detection and precision tests are crucial in the context of this solution, in order to determine the viability of this system, when identifying the room where the visitor is at a specific moment, inside the museum. This correct identification is fundamental in order for the contents of each room to be correctly presented to the visitor. These tests are also useful to determine the best position for beacon placement in a real scenario.

Four sets of experiments were created to evaluate and test the system in all rooms of the museum. The tests were conducted by a researcher, eight Anchor Beacons 2 were used (as described in Section 4.2), as signal emitters, and a Samsung Galaxy Tab S7+ was used as a receiver, equipped with Bluetooth 5.0 (A2DP adapter) and Android 10 operating system. The beacons were configured with an advertising interval signal of 350 ms and a range of 4 m (1 TX power (TX Power: https://support.kontakt.io/hc/en-gb/articles/4413258518930-Beacon-transmission-power-range-and-RSSI (accessed on 21 May 2023))) for border beacons and 10 m (2 TX power) for inside beacons. Border beacons are the ones that are placed near entrances or exits of a room, while inside beacons, as the name indicates, are the ones that are placed inside the rooms.

These two types are needed in order to obtain a good visitor location inside the museum, once the detection of at least one frontier beacon combined with the detection of at least one inside beacon (both representing the same room) allow the App to know the exact room where the user is located. Beacon positioning criteria had to be created in order to obtain the best position scenario—covering all room areas with signals and preventing outsider beacons detection.

### 8.1. Test Scenarios

In each of the four scenarios, beacons were placed, and some positions (Px) were defined that represent the places where measures were taken. The beacons depicted with a blue color (Bx) represent border beacons and were configured with a 4 m range; the beacons depicted with a green color (Bx) represent inside beacons configured with a 10 m range.

The evaluation consisted of capturing the RSSI in each position Px and assessing which beacons were being detected. In each scenario, the route taken always starts in P1, then P2, and so on until the last position of each scenario.

**Figure 7:**
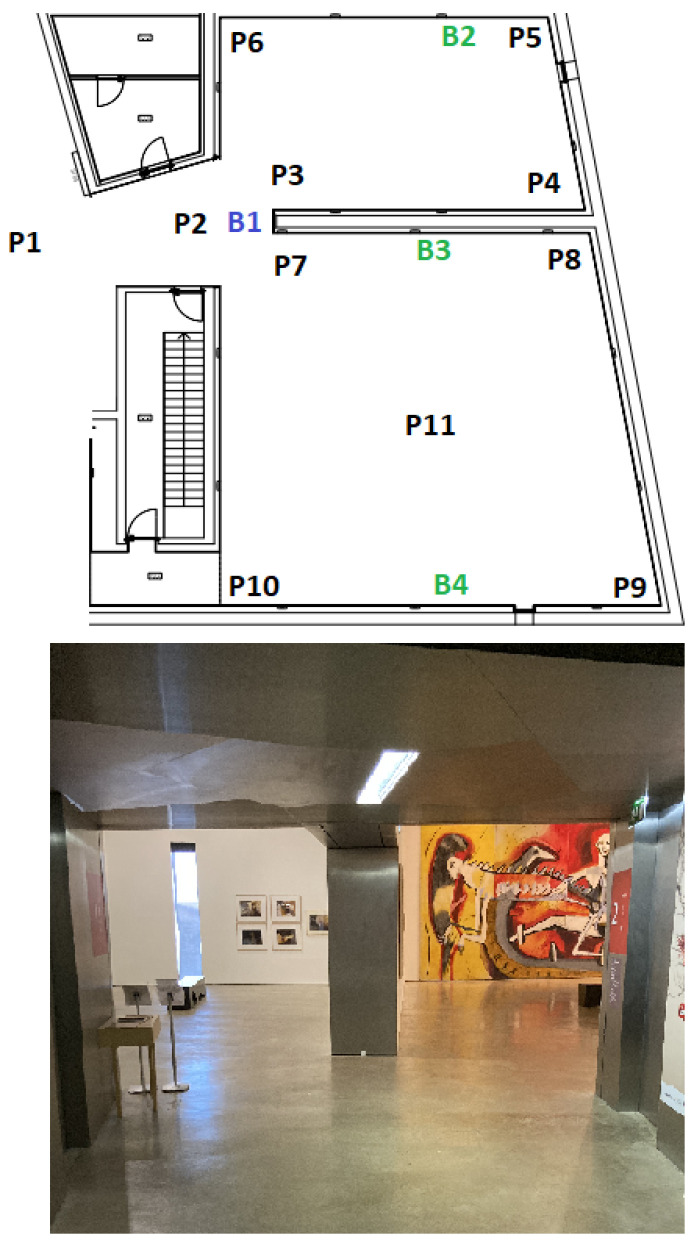
First scenario.

**Figure 8:**
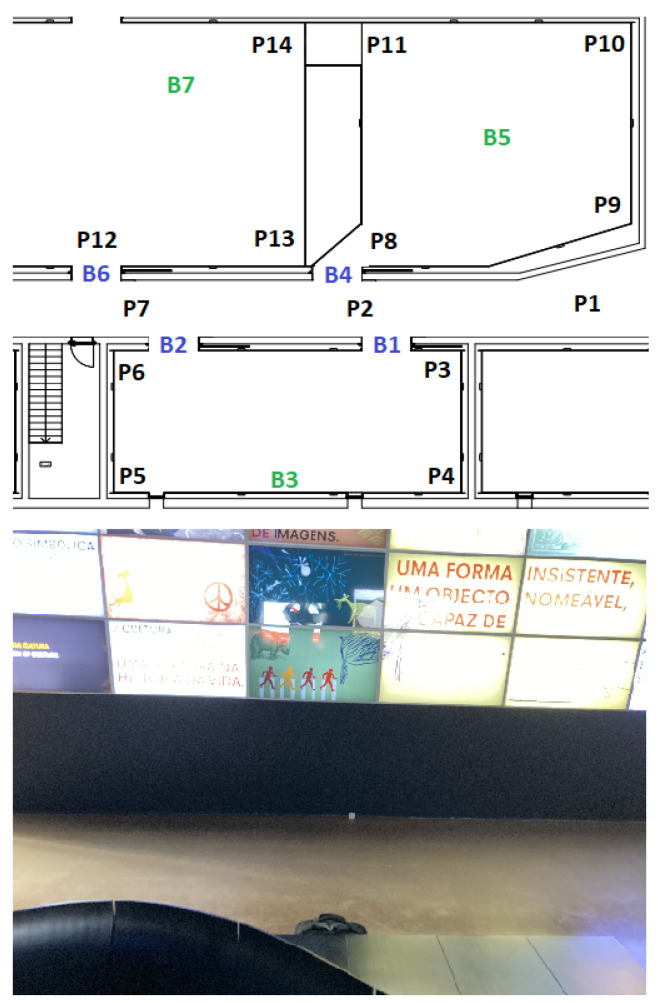
Second scenario.

Figure 7 presents the floorplan at the top side of the image with three inside beacons (B2, B3, and B4), one border beacon (B1), and eleven positions; on the bottom, a real image of the indoor space of the museum is shown. Figure 8 illustrates the second scenario. Again, on the top side of the image, it is possible to see the location of the four border beacons, the three inside beacons, and fourteen test positions.

**Figure 9:**
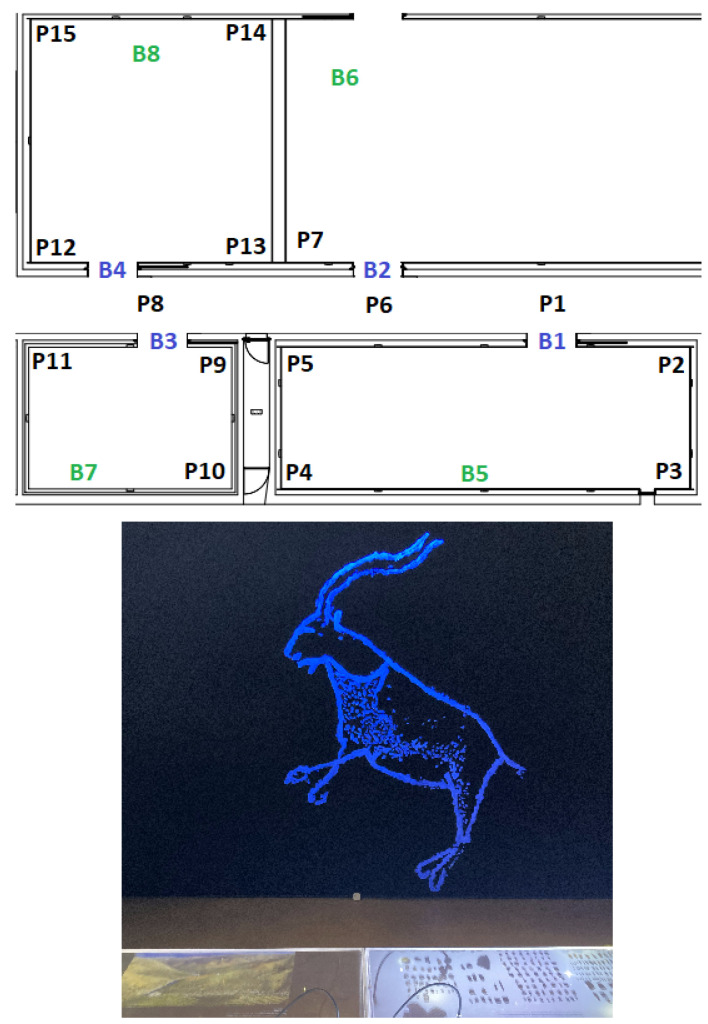
Third scenario.

**Figure 10:**
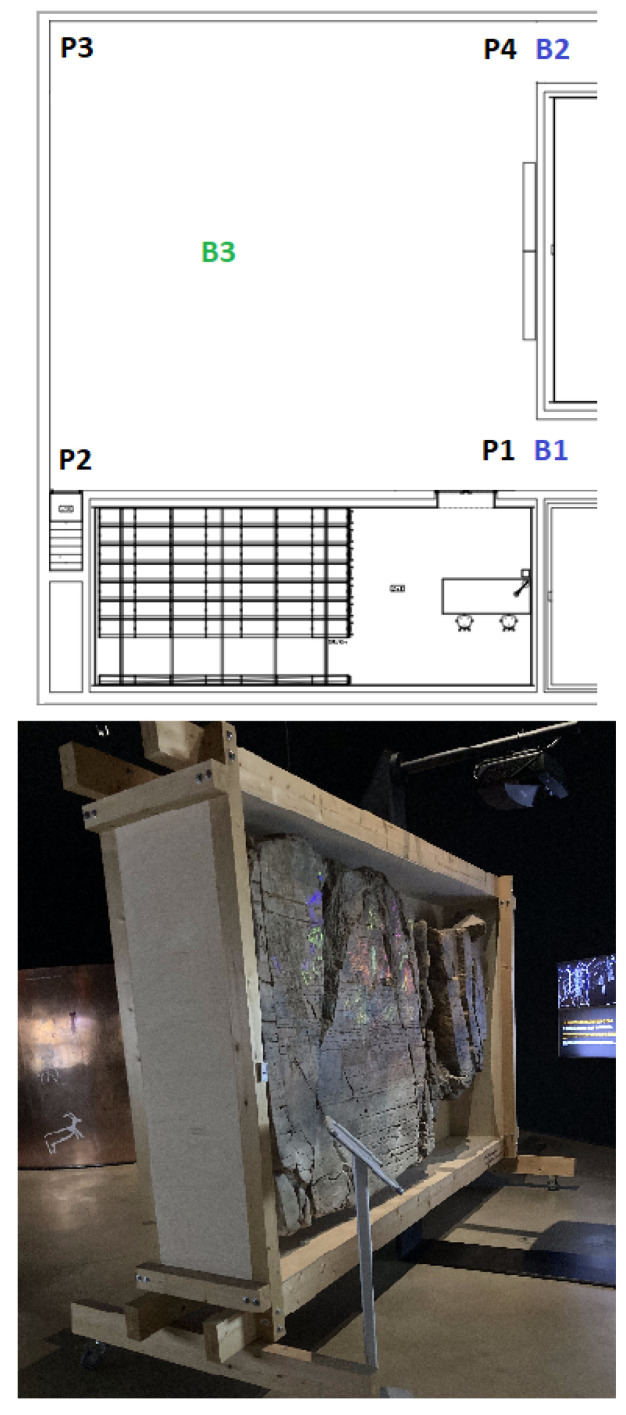
Fourth scenario.

Figure 9 represents the third test scenario that uses four border beacons, four inside beacons, and fifteen test positions in total. The fourth and last scenario is shown in Figure 10 and uses two border beacons, one inside beacon, and four test positions.

### 8.2. Test Results: RSSI Analysis

**Figure 11:**
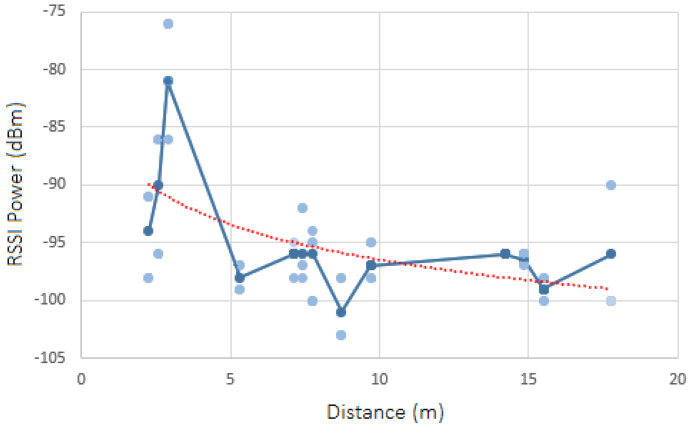
Raw RSSI values, averages, and curve fittings—scenario 1.

**Figure 12:**
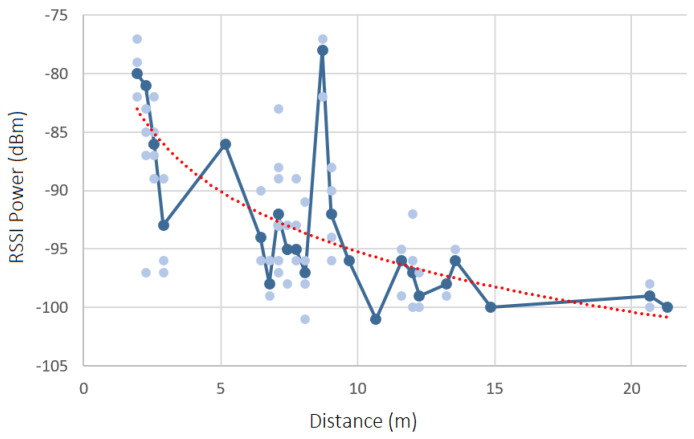
Raw RSSI values, averages, and curve fittings—scenario 2.

**Figure 13:**
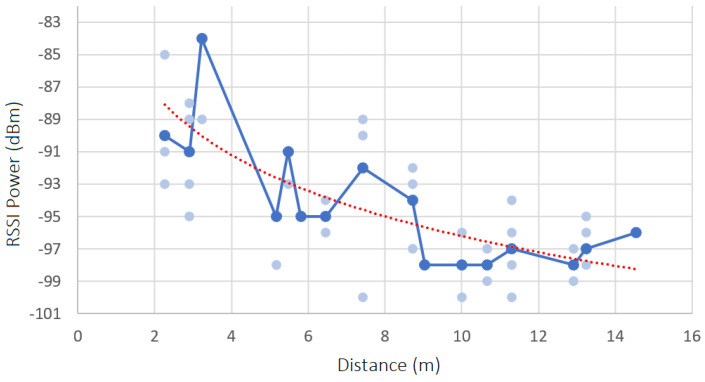
Raw RSSI values, averages, and curve fittings—scenario 3.

**Figure 14:**
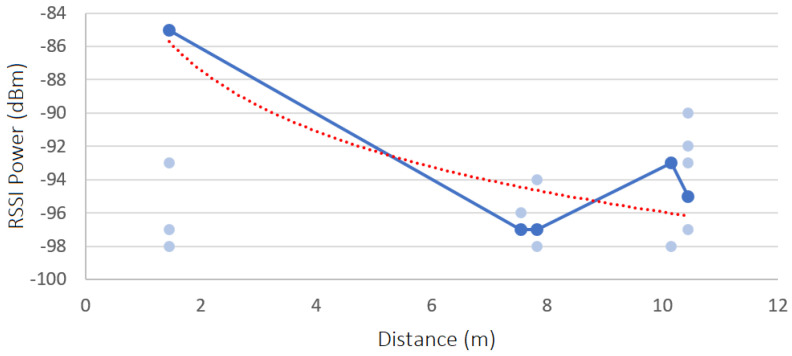
Raw RSSI values, averages, and curve fittings—scenario 4.

In each scenario, the RSSI in each position Px was captured, one time, over a period of 60 s regarding all inside and border beacons. The results are shown in Figure 11, Figure 12, Figure 13 and Figure 14. Each diagram presents the raw RSSI values, the average, and the overall curve fitting. As expected, the RSSI values reduce when the distance increases, as can be seen in all curve fittings, and according also to Figure 2. The average power values do not create a line totally equal to what is expected in theory due to several factors such as magnetic interference, thick walls, and museum contents/artifacts between the devices and the beacons. However, it can be concluded that the further the beacon is positioned, the more reduced the RSSI power that is received.

Thus, if the target museum environment is more or less suitable for this beacon indoor-location technology, the path loss value was calculated for all four scenarios, using the respective formula presented in Section 3.2. For the RSS1 value (RSS value at 1 m from the beacon), two different TX power values (TX1 4 m and TX2 10 m) were used, and the RSS values mean was calculated: (−84−81)/2 = −82.5. The mean RSSI and distance values were also calculated for all scenarios.

#### 8.2.1. Scenario 1—RSSI Analysis

The path loss value obtained for scenario 1 is the following:

- **Path Loss**: 1.397 dBm

#### 8.2.2. Scenario 2—RSSI Analysis

The path loss value obtained for scenario 2 is the following:

- **Path Loss**: 0.908 dBm

#### 8.2.3. Scenario 3—RSSI Analysis

The path loss value obtained for scenario 3 is the following:

- **Path Loss**: 1.349 dBm

#### 8.2.4. Scenario 4—RSSI Analysis

The path loss value obtained for scenario 4 is the following:

- **Path Loss**: 1.234 dBm

#### 8.2.5. Charts’ Findings

All values obtained are relatively low, which means that the museum environment is suitable for a beacon-based indoor location. The four scenarios have similar path loss values, except for scenario 2, which has a lower value, which means it is a better environment to detect the user location with more accuracy, inside the museum. The path loss value is lower in this scenario because it has fewer contents and artifacts inside the rooms and has fewer metal structures, which leads to less magnetic interference.

### 8.3. Test Results: Detection Analysis

The set of carried-out tests also intended to analyze the stability of the received signal, in each of the different positions Px defined in each scenario. This analysis allows us to understand whether the detection is correctly carried out, considering different distances and spaces with different characteristics. The results are presented in Table 3, Table 4, Table 5 and Table 6. Each table presents the various positions and beacons involved in the scenario. The green-colored cells represent the beacons whose signal was correctly received throughout the entire test duration, carried out for 60 s, with detection every 350 ms. The cell also displays the distance from the position to the beacon. The yellow cells represent the beacons whose detection was intermittent throughout the test. The red cells represent beacons that were never detected but should have been detected. Finally, the gray cells represent beacons that were detected but were placed in adjacent rooms. The correct beacon detection has a direct impact on the identification of the space in which the user is located, from which the contents are downloaded and presented.

Excessive beacon detection is neither critical nor problematic since, at the limit, they identify a room that is close to the user. However, missing detection is critical and sometimes results in the wrong identification of the room where the user is located (in the case of beacons from a nearby room not being detected), which causes a delay in the presentation of content to the user. Incorrect detection can occur for several reasons. In this real test scenario, these incorrect detections were due to magnetic interference (many walls and museum contents contain metal structures that allow the Faraday cage effect) and also to museum contents and artifacts existing between the device and the beacons.

**Figure 15:**
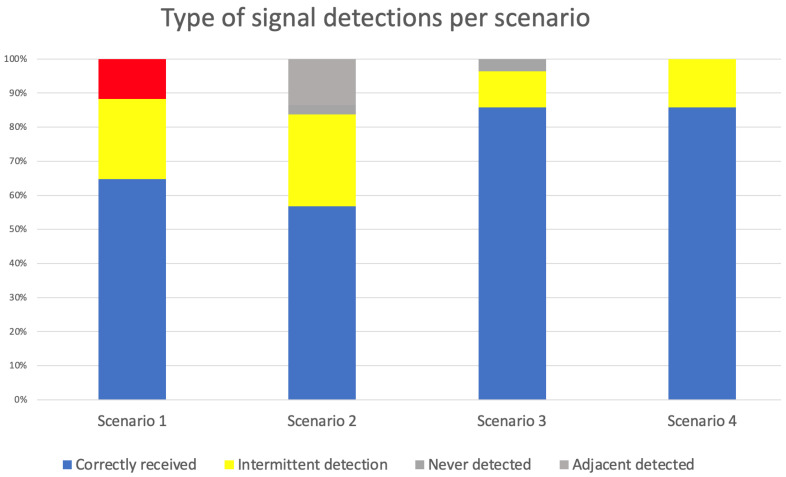
Type of signal detections per scenario.

Table 7 presents a summary of the number of signals received in each scenario, according to their type, which is also presented in a graph format in Figure 15.

### 8.4. Indoor Room Identification

The analysis of the detection of beacons presented in the previous section is essential to the correct identification of the museum room where the user is located, which triggers the presentation of the associated contents in a correct manner.

Based on the detection analysis made in each position Px of each scenario, we were able to calculate the accuracy of the room detection in each scenario and consequently the accuracy of the solution for the Foz Côa Museum. Table 8 shows the results. The average accuracy is approximately 93%.

## 9. Discussion

### 9.1. Pros and Cons—Proposed Solution

This simple beacon centroid approach offers several advantages. Firstly, it provides an intuitive mapping between beacons and rooms, allowing an easy setup and management. Secondly, by not relying on sophisticated RSSI-based techniques, our system is less susceptible to the challenges posed by signal interference, multipath propagation, and variations in signal strength. Moreover, the simplicity of our approach translates into lower hardware requirements, thus it is suitable for reduced computational devices, which increases the range of possible users.

However, it is important to note that this simplified algorithm does not offer the same level of accuracy and precision as advanced RSSI-based algorithms, some using advanced machine learning and neural network-based methods. The range of Bluetooth signals can be influenced by factors such as obstructions, signal attenuation, and environmental conditions. Therefore, while our system provides a viable solution for basic indoor positioning applications, it may not be suitable for scenarios that require highly accurate location information.

The core strength of our solution lies in its flexibility and adaptability, which allow it to accommodate diverse architectural configurations and room dispositions. The primary requirement for scalability is to ensure the optimal positioning of BLE beacons within the building. By strategically placing the beacons based on the specific layout and spatial characteristics of each building, our system can effectively establish a robust indoor localization infrastructure. Moreover, our system’s underlying technology and algorithms are designed to support scalability without requiring modifications to the core architecture. This inherent scalability allows us to seamlessly extend the solution to other buildings with distinct layouts, minimizing implementation complexities and optimizing resource utilization. This extension is already being considered for other museums in Portugal.

During the experimental and validation tests conducted in the real museum scenario, the system exhibited satisfactory performance under normal visitor flow conditions, typically involving up to 10 to 15 visitors within each room. Under these typical occupancy levels, the system demonstrated accurate localization capabilities, successfully identifying and tracking users’ positions within the museum. However, it is essential to acknowledge that the system’s localization performance may be influenced by higher visitor densities, as an increased number of individuals within the museum could lead to higher signal interference and more complex localization challenges. As the occupancy levels rise, the system may encounter greater difficulties in precisely determining individual positions, potentially resulting in a reduction in localization accuracy.

### 9.2. Multiple Beacons Detection

The study aimed to enhance visitor experiences by providing seamless navigation across different exhibit zones. The practical scenario criteria on which the current study was based do not include the necessity to have multiple maps in a room, although there is no technological impediment to this. When the application detects multiple beacons simultaneously, the corresponding zones are considered proximate, resulting in the seamless loading of virtual maps for all these areas. This approach allows visitors to effortlessly transition between exhibit zones and ensured an uninterrupted and immersive museum experience.

Technological constraints were not a limiting factor in this regard. To enable the simultaneous presence of multiple maps for a single, each beacon identifying a specific zone needs to be carefully calibrated to cover the required area precisely without extending beyond its intended boundaries.

### 9.3. Beacon’s Positioning

The lessons learned from the case study developed for the Foz-Côa Museum allowed us to define some major considerations that we used and that can also be used in similar scenarios:

- Place one beacon at each door of a room with a reduced range signal (border beacon)—the range is to be determined in each specific scenario;
- Depending on the room size, one or more beacons should be placed inside the room with a wider range signal (inside beacon)—also to be determined in each scenario. It is important to mention that the inside beacon’s range signal should cover the entire inside area of the room;
- Avoid placing the beacons near metal structures;
- Avoid placing the beacons in places with many contents between the beacon and the receiver device;
- Place the beacons in a higher place, allowing a better signal transmission and avoiding unauthorized access.

**Figure 16:**
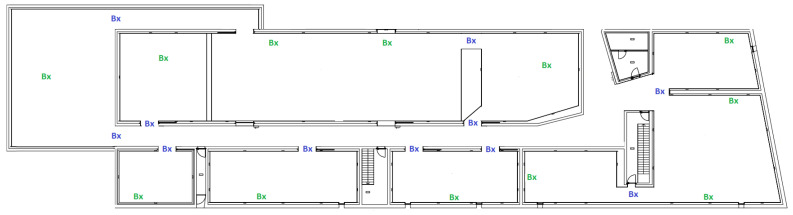
Final proposal for beacons distribution in the Foz Coa Museum.

Based on these general considerations, and after applying them to our case study, we were able to propose a final beacon distribution for the Foz-Côa museum, which is presented in Figure 16. This final distribution solution has a total of 23 beacons, of which 11 are border beacons (configured with a 4 m range) and the other 12 are inside beacons (configured with a 10 m range). With this beacon placement, the full museum area is covered with the smallest amount of beacon devices possible.

## 10. Conclusions

In this paper, an indoor content delivery solution based on BLE beacons and augmented reality was presented, specifically for the Foz Côa Museum in Portugal. A study on BLE beacon technology was presented as a way to support the indoor location, as well as mathematical formulas regarding the RSSI, distance, and path loss. The system’s solution architectures were also presented, more specifically the system’s overview, technological architecture, application workflow, and mobile application architecture. In order to test the effectiveness of BLE beacons in a real environment, four scenarios were created in the Foz Coa Museum. RSSI analysis, beacon’s precision, and accuracy of room detection, for all four scenarios, were presented as experimental results in the real environment. The path loss analysis of all four scenarios is relatively low, which means that the museum environment is suitable for a beacon-based indoor location. The path loss value is lower in one of the scenarios because of the fewer contents and artifacts and fewer metal structures. The beacon detection analysis presented 76% of correctly received signals, 20% of intermittent signals, and 4% of never detected signals. Based on these numbers, the final accuracy of the beacon distribution solution proposed for the Foz Coa Museum achieved a 96% accuracy. Based on the gathered results, the solution proposed in this article is feasible and can be easily replicated in other environments. As a future work, augmented reality content will be developed so they can be offered to visitors during their visit, automatically based on their inferred position.

## Figures and Tables

**Table 1:** Indoor positioning technologies basic specifications.

Indoor Positioning Technology	Range	Accuracy Error	Cost	Power Consumption	Extra Device on User-Side
UWB	Up to 200 m	6–10 cm	High	Low	Yes
Infrared	Up to 30 m	1–2 m	Moderate	Low	Yes
RFID	Up to 10 m	1–3 m	Moderate	Low	Yes
WiFi	Up to 100 m	1–5 m	Low	High	No
BLE Beacon	Up to 100 m	1–5 m	Low	Low	No

**Table 2:** Kontakt anchor beacon 2 specifications.

Connectivity	Bluetooth Low Energy 5.0 (BLE 5.0)
**Range**	Up to 100 m
**Transmission power levels**	−20 to +4 dBm
**Batteries number**	2 (replaceable)
**Battery lifetime**	+8 years
**Micro-controller**	nRF52832
**Dimensions**	49 mm × 49 mm × 15 mm
**Weight**	38 g

**Table 3:** First scenario—signal stability test.

	B1	B2	B3	B4	Correct Room Detection?
**P1**	-	-	-	-	Yes
**P2**	2.58 m		-	17.77 m	Yes
**P3**	1.83 m	9.69 m	-	-	Yes
**P4**	-	7.11 m	-	-	Yes
**P5**	-	2.91 m	-	-	Yes
**P6**	7.43 m	8.72 m	-		Yes
**P7**	2.26 m	-	8.38 m	14.86 m	Yes
**P8**	-	-	5.33 m	14.21 m	Yes
**P9**	-	-	15.50 m	7.43 m	Yes
**P10**	-	-	15.50 m	7.76 m	Yes

**Table 4:** Second scenario—signal stability test.

	B1	B2	B3	B4	B5	B6	B7	Correct Room Detection?
**P1**	5.17 m	-	-	6.78 m	-	-	-	Yes
**P2**	2.26 m	-	-	2.26 m	-	-	-	Yes
**P3**	2.58 m	13.24 m	7.43 m	7.11 m	-	-	-	-
**P4**	-	-	-	-	-	-	-	Yes
**P5**	13.57 m	7.75 m	4.58 m	-	-	-	-	Yes
**P6**	2.58 m	2.26 m	9.69 m	-	-	5.17 m	-	Yes
**P7**	-	2.58 m	11.63 m	-	-	2.58 m	11.95 m	Yes
**P8**	-	-	-	13.24 m	6.47 m	-	12.27 m	Yes
**P9**	-	-	-	14.24 m	7.11 m	-	20.67 m	Yes
**P10**	-	-	-	-	7.11 m	-	21.32 m	No
**P11**	-	-	-	-	7.43 m	17.89 m	10.66 m	No
**P12**	-	-	-	-	-	1.94 m	8.72 m	Yes
**P13**	6.65 m	-	-	-	-	8.08 m	9.04 m	Yes
**P14**	-	-	-	-	-	14.86 m	5.17 m	Yes

**Table 5:** Third scenario—signal stability test.

	B1	B2	B3	B4	B5	B6	B7	B8	Correct Room Detection?
**P1**	2.26 m	-	-	-	9.04 m	-	-	-	No
**P2**	5.81 m	-	-	-	11.31 m	-	-	-	Yes
**P3**	8.72 m	-	-	-	10.01 m	-	-	-	Yes
**P4**	14.54 m	-	-	-	8.72 m	-	-	-	Yes
**P5**	-	-	-	-	10.66 m	-	-	-	Yes
**P6**	10.04 m	-	7.43 m	-	-	-	-	-	Yes
**P7**	-	-	-	-	-	-	-	-	-
**P8**	-	-	2.26 m	2.26 m	-	-	-	12.92 m	No
**P9**	-	-	2.91 m	-	-	-	8.72 m	-	Yes
**P10**	-	-	7.43 m	-	-	-	6.46 m	-	Yes
**P11**	-	-	5.49 m	-	-	-	6.14 m	-	Yes
**P12**	-	-	7.43 m	3.23 m	-	-	-	11.31 m	Yes
**P13**	-	-	-	5.81 m	-	-	-	11.31 m	Yes
**P14**	-	-	-	-	-	-	-	5.49 m	Yes
**P15**	-	-	-	13.24 m	-	-	-	5.17 m	Yes

**Table 6:** Fourth scenario—signal stability test.

	B1	B2	B3	Correct Room Detection?
**P1**	1.45 m	-	10.15 m	Yes
**P2**	13.92 m	-	7.83 m	Yes
**P3**	-	-	7.54 m	Yes
**P4**	-	1.45 m	10.44 m	Yes

**Table 7:** Summary of stability tests in all four scenarios.

	Correctly Received	Intermittent Detection	Never Detected	Adjacent Detected
**Scenario 1**	11	4	2	0
**Scenario 2**	21	10	1	5
**Scenario 3**	24	3	1	0
**Scenario 4**	6	1	0	0

**Table 8:** Room detection accuracy per scenario.

	# Positions	Accuracy
**Scenario 1**	9	100%
**Scenario 2**	13	85%
**Scenario 3**	14	86%
**Scenario 4**	4	100%

## Abbreviations

The following abbreviations are used in this manuscript:

MDPI	Multidisciplinary Digital Publishing Institute
BLE	Bluetooth Low Energy
RSSI	Received Signal Strength Indicator
RF	Radio Frequency
US	Ultrasonic
CPU	Central Process Unit
HVAC	Heating, Ventilating and Air Conditioning

## References

[1] Jiwane A., Khan A.F. Interactive museums: Empowering visitors’ engagement. Proceedings of the 3rd Smart Cities Symposium (SCS 2020).

[2] Vena A., Illanes I., Alidieres L., Sorli B., Perea F. RFID based Indoor Localization System to Analyze Visitor Behavior in a Museum. Proceedings of the 2021 IEEE International Conference on RFID Technology and Applications (RFID-TA).

[3] Handojo A., Lim R., Octavia T., Anggita J.K. Museum Interactive Information Broadcasting Using Indoor Positioning System and Bluetooth Low Energy: A Pilot Project on Trowulan Museum Indonesia. Proceedings of the 2018 3rd Technology Innovation Management and Engineering Science International Conference (TIMES-iCON).

[4] Duchetto F.D., Baxter P., Hanheide M. Lindsey the Tour Guide Robot—Usage Patterns in a Museum Long-Term Deployment. Proceedings of the 2019 28th IEEE International Conference on Robot and Human Interactive Communication (RO-MAN).

[5] Alletto S., Cucchiara R., Del Fiore G., Mainetti L., Mighali V., Patrono L., Serra G. (2016). An Indoor Location-Aware System for an IoT-Based Smart Museum. IEEE Internet Things J..

[6] Verde D., Romero L., Faria P.M., Paiva S. Architecture for Museums Location-Based Content Delivery using Augmented Reality and Beacons. Proceedings of the 2022 IEEE International Smart Cities Conference (ISC2).

[7] Spachos P., Papapanagiotou I., Plataniotis K.N. (2018). Microlocation for Smart Buildings in the Era of the Internet of Things: A Survey of Technologies, Techniques, and Approaches. IEEE Signal Process. Mag..

[8] Sadowski S., Spachos P. (2018). RSSI-Based Indoor Localization with the Internet of Things. IEEE Access.

[9] Pivato P., Palopoli L., Petri D. (2011). Accuracy of RSS-Based Centroid Localization Algorithms in an Indoor Environment. IEEE Trans. Instrum. Meas..

[10] Casareo K., Chaczko Z. Beacon-Based Localization Middleware for Tracking in Medical and Healthcare Environments. Proceedings of the 2018 12th International Symposium on Medical Information and Communication Technology (ISMICT).

[11] Jahan K., Adler S., Ahmad J., Hansen C. Wireless Tracking for Industrial Services. Proceedings of the 2018 15th Workshop on Positioning, Navigation and Communications (WPNC).

[12] Giuliano R., Cardarilli G.C., Cesarini C., Di Nunzio L., Fallucchi F., Fazzolari R., Mazzenga F., Re M., Vizzarri A. (2020). Indoor Localization System Based on Bluetooth Low Energy for Museum Applications. Electronics.

[13] Spachos P., Plataniotis K.N. (2020). BLE Beacons for Indoor Positioning at an Interactive IoT-Based Smart Museum. IEEE Syst. J..

[14] Pušnik M., Galun M., Šumak B. (2020). Improved Bluetooth Low Energy Sensor Detection for Indoor Localization Services. Sensors.

[15] Shum L.C., Faieghi R., Borsook T., Faruk T., Kassam S., Nabavi H., Spasojevic S., Tung J., Khan S.S., Iaboni A. (2022). Indoor Location Data for Tracking Human Behaviours: A Scoping Review. Sensors.

[16] Duong N.S., Nguyen T.P., Nguyen Q.T., Dinh-Thi T.M. (2023). Improving indoor positioning system using weighted linear least square and neural network. Int. J. Sens. Netw..

[17] Labinghisa B.A., Lee D.M. (2021). Neural network-based indoor localization system with enhanced virtual access points. J. Supercomput..

[18] Maduranga M., Abeysekera R. (2022). Bluetooth low energy (ble) and feed forward neural network (ffnn) based indoor positioning for location-based iot applications. Int. J. Wirel. Microw. Technol. (IJWMT).

[19] Filippoupolitis A., Oliff W., Loukas G. Bluetooth Low Energy Based Occupancy Detection for Emergency Management. Proceedings of the 2016 15th International Conference on Ubiquitous Computing and Communications and 2016 International Symposium on Cyberspace and Security (IUCC-CSS).

[20] Tekler Z.D., Low R., Yuen C., Blessing L. (2022). Plug-Mate: An IoT-based occupancy-driven plug load management system in smart buildings. Build. Environ..

[21] Balaji B., Xu J., Nwokafor A., Gupta R., Agarwal Y. (2013). Sentinel: Occupancy Based HVAC Actuation Using Existing WiFi Infrastructure within Commercial Buildings. Proceedings of the 11th ACM Conference on Embedded Networked Sensor Systems, SenSys ’13.

[22] Tekler Z.D., Chong A. (2022). Occupancy prediction using deep learning approaches across multiple space types: A minimum sensing strategy. Build. Environ..

[23] Torres-Solis J., Falk T., Chau T. (2010). A Review of Indoor Localization Technologies: Towards Navigational Assistance for Topographical Disorientation.

[24] Gu Y., Lo A., Niemegeers I. (2009). A survey of indoor positioning systems for wireless personal networks. IEEE Commun. Surv. Tutor..

[25] Botler L., Spörk M., Diwold K., Römer K. Direction Finding with UWB and BLE: A Comparative Study. Proceedings of the 2020 IEEE 17th International Conference on Mobile Ad Hoc and Sensor Systems (MASS).

[26] Tekler Z.D., Low R., Gunay B., Andersen R.K., Blessing L. (2020). A scalable Bluetooth Low Energy approach to identify occupancy patterns and profiles in office spaces. Build. Environ..

[27] Potortì F., Park S., Jiménez Ruiz A.R., Barsocchi P., Girolami M., Crivello A., Lee S.Y., Lim J.H., Torres-Sospedra J., Seco F. (2017). Comparing the Performance of Indoor Localization Systems through the EvAAL Framework. Sensors.

[28] Kalbandhe A., Patil S. Indoor Positioning System using Bluetooth Low Energy. Proceedings of the 2016 International Conference on Computing, Analytics and Security Trends (CAST).

[29] Schjørring A., Cretu-Sircu A.L., Rodriguez I., Cederholm P., Berardinelli G., Mogensen P. (2022). Performance Evaluation of a UWB Positioning System Applied to Static and Mobile Use Cases in Industrial Scenarios. Electronics.

[30] Di Pietra V., Dabove P. Recent advances for UWB ranging from Android Smartphone. Proceedings of the 2023 IEEE/ION Position, Location and Navigation Symposium (PLANS).

[31] Lee Ann B. (2022). Advantages and Disadvantages of Beacon Technology. https://leeannbaugh.com/advantages-and-disadvantages-of-beacon-technology/.

[32] Shang F., Su W., Wang Q., Gao H., Fu Q. (2014). A Location Estimation Algorithm Based on RSSI Vector Similarity Degree. Int. J. Distrib. Sens. Netw..

[33] Kochláň M., Miček J., Ševčík P. 2.4 GHz ISM band radio frequency signal indoor propagation. Proceedings of the 2014 Federated Conference on Computer Science and Information Systems.

